# Accuracy of whole slide image based image analysis is adversely affected by preanalytical factors such as stained tissue slide and paraffin block age

**DOI:** 10.1016/j.jpi.2022.100121

**Published:** 2022-06-28

**Authors:** Nada Shaker, Ruhani Sardana, Satoshi Hamasaki, David G. Nohle, Leona W. Ayers, Anil V. Parwani

**Affiliations:** a Department of Pathology, The Ohio State University Wexner Medical Center, Columbus, OH, USA; b Cooperative Human Tissue Network (CHTN) Midwestern Division (MWD) State University (OSU), Columbus, OH, USA

**Keywords:** Whole slide images, Tumor area identification, Slide age, Overall tumor percentage, AI algorithm

## Abstract

**Background:**

Personalized medicine and accurate quantification of tumor and biomarker expression have become the cornerstone of cancer diagnostics. This requires Quality Control (QC) of research tissue samples to confirm adequate targeted tumor tissue sampling. Digitalization of stained tissue slides offer a precious way to archive, preserve, and retrieve necessary information when needed. This study is aimed to assess the most significant pre-analytic and analytic factors that might contribute to the efficacy of obtaining accurate whole slide images (WSIs) interpretation. Various studies are needed to identifysuch factors to allow for appropriate AI application and adequate tumor area/percentage quantification.

**Methods:**

Hematoxylene and Eosine (H&E) satined WSIs collected from tissue specimens provided by the Cooperative Human Tissue Network (CHTN) Midwestern Division (CHTNMWD) were analyzed. Tissue specimens were processed, fixed, stained, and scanned contemporaneously (within 1 month). Two cohorts of malignant, colorectal cancer, 20X WSI (ScanscopeXT, Leica Biosystems, Illinois), were assembled. The study identified a "recent cohort" that included 76 WSIs created on 2018 or later. "Aged cohort" included 73 WSIs from specimens procured in the period of (2012-2014). Twenty recent WSIs of adenocarcinoma cases were used to construct WSIs analysis algorithms (VIS, Visiopharm A/S, Denmark) using machine learning to produce morphometric maps and calculate tissue and tumor areas.

**Results:**

Algorithmic analysis of 69 WSIs from rescanned aged slides vs. that of contemporaneous WSIs concluded 18 (28%) similar finding in tumor areas (within 10%), 56 (82%) had identicaltissue areas, and 54 (79%) had similar tumor percentages.

**Conclusion:**

WSIs of aged H&E slides and stained paraffin block re-cuts produce different tumor quantification compared to those of original scanned sslides most likely due to pre-analytical factors. The difference in tumor area detected between original and rescanned WSIs trended upward in the period between 2012 and 2014. Less tumor area was detected as the slides age. Recut and H&E-stained tissues from stored paraffin blocks may detect more tumor due to excess eosinophilia. These results highlights the value of documenting archives of H&E WSIs collected at the procurement time. Such images provide a superior archive over glass slides and Formalin-Fixed Paraffin-Embedded (FFPE) blocks and contribute betterg to WSIs analysis application.

## Introduction

Digital pathology promises a high standard quantity and quality tissue analysis. The process of tissue digitization includes 4 sequential parts: image acquisition (scanning), storage, editing, and images display.^1^ Digital patholoy brings along a whole gamut of benefits with the most powerful and practical being an easier method to archive, store, and retrieve images. Personalized medicine, accuracy, and precision to grade tumors and quantify biomarker expression have become the cornerstone for rendering a final diagnosis. This raises the need for frequent quality check and validation of methods. Quality control (QC) of cancer research samples require review by a pathologist to assure that adequate targeted tumor presence. Digital or machine-based computational diagnostic pathology is a dynamic reality for QC and is expanding with the implementation of whole slide images (WSIs) analysis. The College of American Pathologists Pathology and Laboratory Quality Center provide validation criteria for diagnostic WSIs management purposes.^2^

This study is aimed to identify the role of pre-analytic and analytic factors^3^^,^^4^^,^^5^ in quantifying tumor percentage and answer the following question: Can several factors such as digital image reproducibility, different machine algorithms, slide age, and hematoxylin and eosin (H&E) stained slide condition be mitigated to allow better morphometric algorithms tumors quantification?^6^ The application of such diagnostic algorithms will likely drive conformity of tissue variables. Digital pathology offers potential computer-aided diagnosis, significantly reducing the pathologists’ workload and paving the way for accurate prognostication with reduced inter- and intra-observer variations. Digital pathology and the application of AI is attracting the attention of pathologists worldwide, also drawing the attention of young medical students to choose pathology as a future career.^7^

An important point to be addresses is that measuring high tumor mutational burden (TMB-H) remains challenging due to the difficulty of obtaining adequate tissue material from certain cancer types such as non-small cell lung cancers. Up to this date, no data has supported the possibility of using cell blocks (CBs) for TMB evaluation; therefore, evaluation of the feasibility of analyzing TMB on CBs is also necessary.^8^

The US National Cancer Institute funds the CHTN to provide high-quality human tissues for translational research. The Midwestern Division is 1 of 6 that serve the investigators in the USA and Canada.^9^^,^^10^

## Methods

We used quantitative data generated with the above morphometric algorithm to graph 31 individual tissue cases with numerical data for tumor area and tumor percentage. The 3 cohorts used were: (1) WSIs from tissue procured in the period of (2012-2014), (2) re-scanned "same stored glass WSIs", and (3) tissue re-cut and H&E staining from the stored paraffin block. 20X WSIs were produced by Scanscope XT, Leica Biosystems, Buffalo Grove, IL, USA. H&E QC slides with whole slide images (WSIs) from the MWD Image Repository were utilized.^11^^,^^12^ Specimens were processed, fixed, stained, and scanned contemporaneously (within 1 month). Two cohorts of malignant, colorectal cancer, 20X WSI (ScanscopeXT, Leica Biosystems, Illinois) and slides were assembled. "Recent cohort" included 76 cases collected in 2018 or later. "Aged cohort" cohort included 73 specimens that has been procured in the period of (2012-2014). Twenty recent WSIs of adenocarcinomas were used to construct image analysis algorithms (VIS, VisiopharmA/S, Denmark) using machine learning to produce morphometric maps and calculate tissue and tumor areas.

Special samples collections and documentation respected the regulatory regarding digital processing, and web archiving. Thus, patients’ identifications and protected personal health information was secured.

## Results

WSIs of aged H&E slides produced different tumor quantification using a morphometric algorithm (VIS, Visiopharm A/S, Hørsholm, Denmark) when compared to the original WSIs. This might be due to different pre-analytical and analytical factors. The difference in the tumor area and tumor percentage detected between the original and the rescanned images trended upward from 2012 to 2014. Less tumor area was detected as glass slides and paraffin blocks aged.

**Fig. 1:**
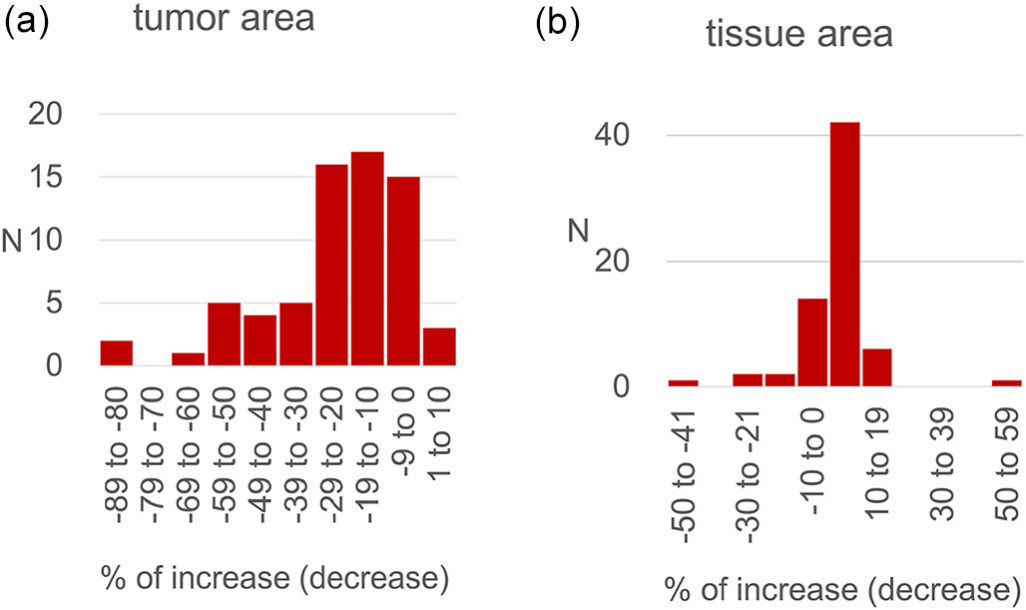
Algorithmic analysis results of 68 images (excluding 1 that had developed a bubble due to loosened cover slip) from re-scanning aged slides vs. that of original images found 18 (26%) had similar tumor areas (within 10%), 56 (82%) similar tissue area, and 54 (79%) similar % tumor.

**Fig. 2:**
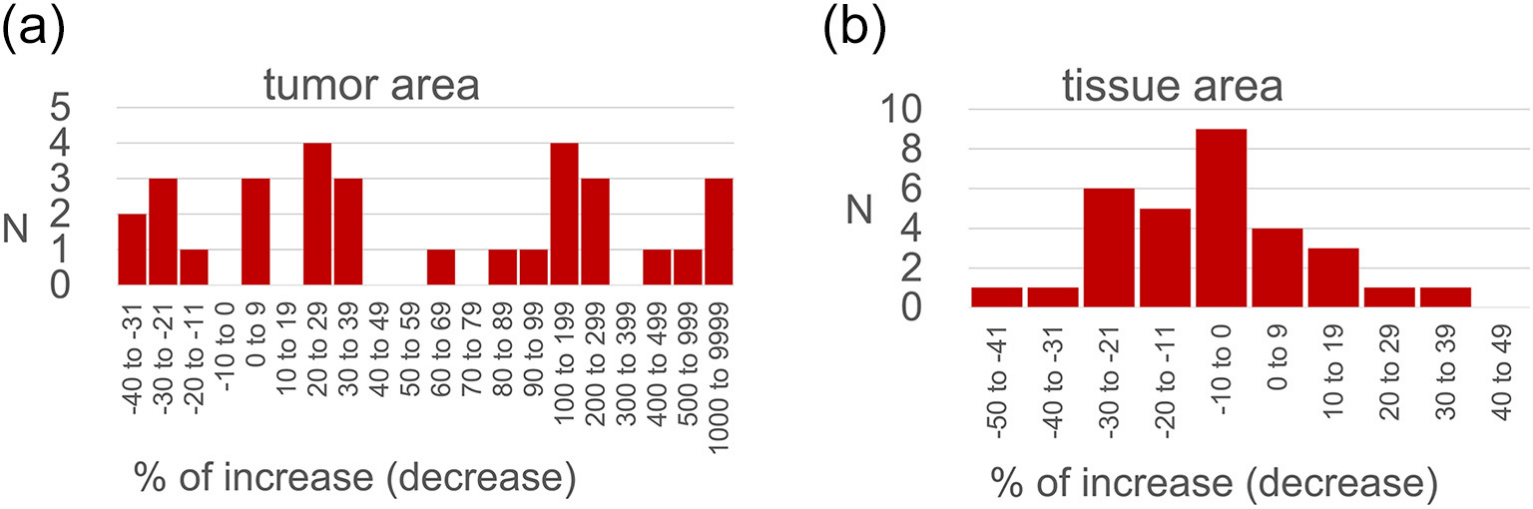
Algorithmic analysis results of 31 images from re-cutting aged blocks vs. that of left/label end section of contemporaneous images found 3 (10%) had similar tumor areas (within 10%), 13 (42%) similar tissue area, and 9 (29%) similar % tumor.

**Fig. 3:**
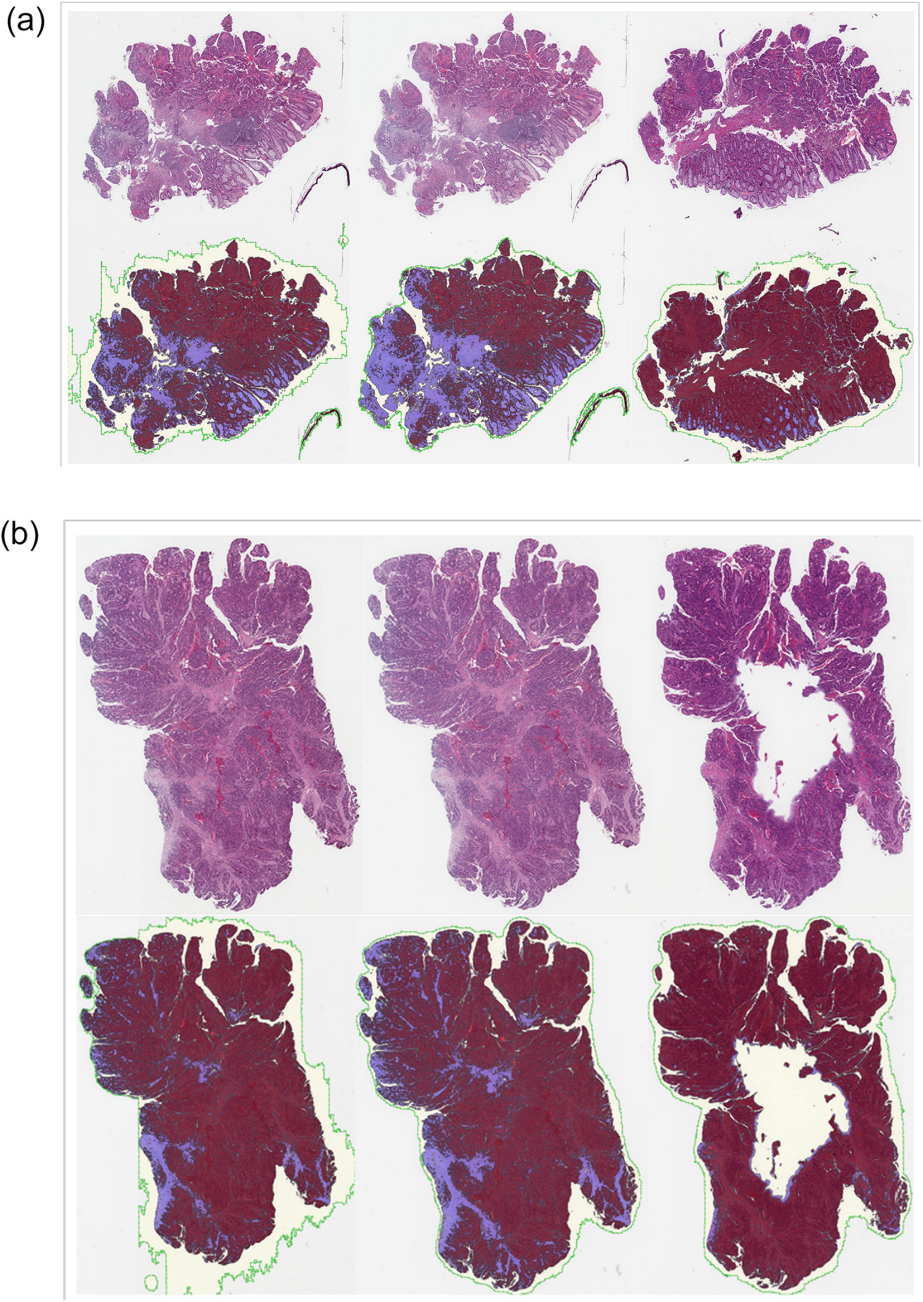
(a) Left to right: original, re-scan, re-cut (with “chatter”). Top row: H&E image, bottom row: classification maps (M1130655A). (b) Left to right: original, re-scan, re-cut (with tissue fallout). Top row: H&E image, bottom row: classification maps. (M3140080A).

WSIs of re-scanned stored glass slides showed less accuracy in identifying tissue with reduced detection of tumor area and overall tumor percentage compared to other 2 cohorts (Fig. 1, Fig. 2). Only 32% WSIs of re-cut tissue sections were histologically similar to those of the original tissue. Significant tissue “chatter”, folds and wrinkles, so called pre-analytic factors, were identified in 45% of sections. (Fig. 3a). 32% of sections showed irregular holes with missing tissue (“fallout”). (Fig. 3b). The algorithm calculated an excessive outline of tumor area when compared to tumor area positioning in the original tumor slides. An essential analytic factor that highly contribute to a successful algorithmic expected performance.

**Fig. 4:**
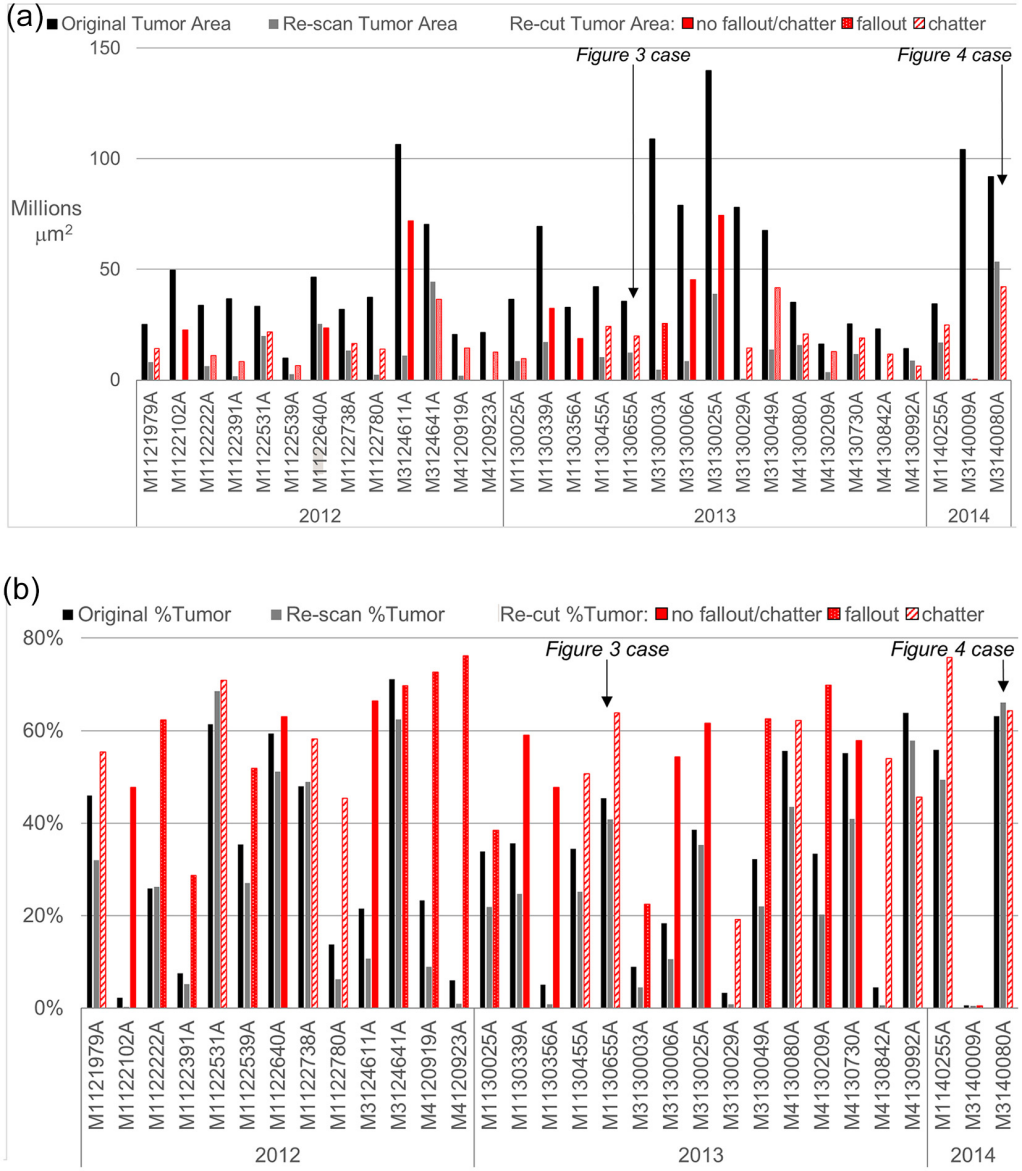
(a) Comparison of size of tumor area measured in original vs. re-scan vs. re-cut by case; tissue fallout and chatter indicated for re-cuts. (b) Comparison of percent tumor measured in original vs. re-scan vs. re-cut by case; tissue fallout & chatter indicated for re-cuts.

The paraffin block re-cut WSIs calculated a significantly lesser tumor area. In 26 cases, the tumor percentage in the re-cut WSIs significantly exceeded the tumor percentage identified in other cohorts (Fig. 4a, 4b).

Algorithmic analysis results of 68 WSIs (excluding one that had developed a bubble due to a loosened cover slip) from re-scanned aged slides vs. that of original WSIs concluded 18 (26%) with similar tumor areas (within 10%), 56 (82%) with similar tissue area, and 54 (79%) with similar tumor percentage (Fig. 1).

Algorithmic analysis results of 31 WSIs from re-cutting aged blocks vs. that of left/label end section of contemporaneous WSIs found 3 (10%) had similar tumor areas (representing 10%), 13 (representing 42%) similar tissue area, and 9 (consisting of 29%) similar tumor percentage (Fig. 2).

In general, tumor area was decreased in re-scanned WSIs when compared to the WSIs obtained from the original slides.

Less tumor area was detected as the slides aged. However, tumor area was greatly increased in re-cut and re-stained slides compared to the original slides. This might be explained by the theory of the identification of more tumor tissue sections on the re-cuts, or it might be related to the fact that the algorithm was able to detect more true-positive tumor tissues on the re-cut samples.

In our study, no nuclear size calculation on re-cuts was performed, however it is important to mention that nuclear size calculation may provide an important internal control to evaluate.

**Fig. 5:**
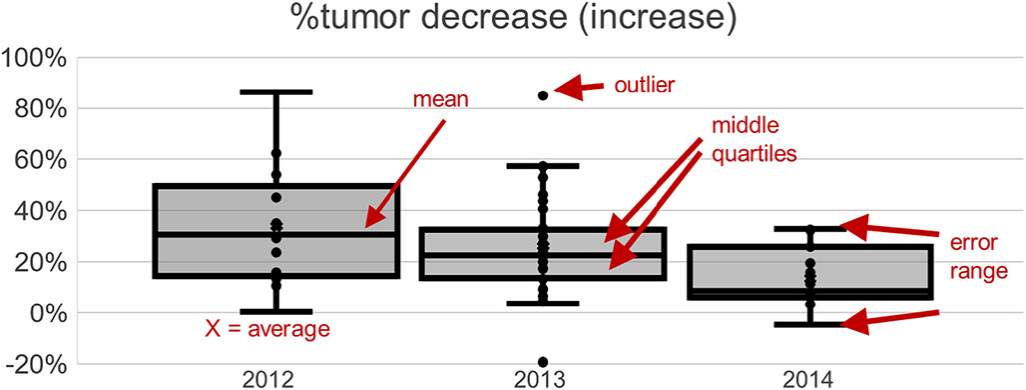
Comparing re-scan vs. original images for 31 cases that have analysis metrics: 26 cases increased up to 1998%; 12 cases increased more than 100%; tissue area changes are within 30% in 30 cases.

Of 31 cases, 26 cases increased up to 1889% and 12 cases increased more than 100% although tissue area changes are within 30% in 30 cases (Fig. 5).

## Discussion

Our finding suggests that 1 month of tissue processing and hematoxylin and eosin staining (H&E staining) is the ideal period for obtaining a most accurate and reproducible morphometric quantification of the slide. Thus, the original scan cohort performed best. For centuries, H&E-stained slides has been the most common approach to reach a final diagnosis. However, the storage of the H&E slides for long period can be challenging. Previously published literature has demonstrated that paraffin tissue blocks stored at room temperature beyond 2 years have significant loss of RNA–DNA on assay with likely accumulation of nucleic acid as a break down product.^13^ This appears supported by our observation of acidification of tissues on H&E staining after 2 years. Another way of retrieval can be to perform re-cuts on the preserved paraffin blocks. This holds its own limitations. Serial sectioning may cut through the area of interest and moreover may exhaust the tissue with area of interest.^14^ The latter becomes crucial with smaller core needle biopsies. Such precious samples require the utmost accuracy and caution while processing to avoid providing potential diagnostic pitfalls.^15^ A worth to mention is the importance of the identification of the ideal display for WSIs analysis.^16^ Various artificial intelligence-based commercial software solutions for pathologists are available in order to augment digital pathology performance.^17^

Our study concludes that the fresh-scanned slides tissues provide the most consistent results for morphometric quantifications.^18^ Tissue pre-analytical and analytical factors do influence the performance of tissue morphometric analysis for tumor quantification. In summary, pre-analytic factors such as re-stained and re-cut tissue slides and paraffin block age in addition to an accurate algorithm tumor outline most significantly contributed to the differences between the cohorts.^19^

The WSI morphometric application tends to incorrectly estimate tumor areas in the aged re-scanned slides and paraffin block re-cuts compared to the original WSI of freshly prepared tissue samples. Also, variable outlines for tissue areas were identified in our cohorts. Therefore, appropriate slides selection to optimize the tumor area outlines identification is necessary to obtain accurate results.

Further investigation is needed to define other analytical factors that might contribute to the variation and therefore inadequate tumor detection in the aged-stored slides and paraffin block re-cuts and stains. WSIs obtained from fresh prepared tissue are the best source for QC of research tissues.

## Conclusions

The awareness of the contributing pre-analytical factors of the quality of WSIs is important to obtain a successful AI algorithm application. Critical pre-analytic factors such as tissue preservation criteria, slide age, and preparation process play a significant role in tumor area identification and the overall tumor percentage calculation. We recommend the usage of WSIs obtained from freshly processed tissue to provide the best WSIs for AI algorithm preparation and research study applications. Additional studies to identify other contributing factors and to investigate the appropriate given slide age period is required for quality control research purposes.

## Conflict of interest

The authors declare that they have no known competing financial interests or personal relationships that could have appeared to influence the work reported in this paper.
